# Embodied Cognition: Taking the Next Step

**DOI:** 10.3389/fpsyg.2012.00582

**Published:** 2012-12-28

**Authors:** Roel M. Willems, Jolien C. Francken

**Affiliations:** ^1^Donders Institute for Brain, Cognition and Behaviour, Radboud University NijmegenNetherlands; ^2^Max Planck Institute for PsycholinguisticsNijmegen, Netherlands

**Keywords:** embodied, cognition, semantics, embedded cognition, hypothesis generation

## Abstract

Recent years have seen a large amount of empirical studies related to “embodied cognition.” While interesting and valuable, there is something dissatisfying with the current state of affairs in this research domain. Hypotheses tend to be underspecified, testing in general terms for embodied versus disembodied processing. The lack of specificity of current hypotheses can easily lead to an erosion of the embodiment concept, and result in a situation in which essentially any effect is taken as positive evidence. Such erosion is not helpful to the field and does not do justice to the importance of embodiment. Here we want to take stock, and formulate directions for how it can be studied in a more fruitful fashion. As an example we will describe few example studies that have investigated the role of sensori-motor systems in the coding of meaning (“embodied semantics”). Instead of focusing on the dichotomy between embodied and disembodied theories, we suggest that the field move forward and ask *how and when* sensori-motor systems and behavior are involved in cognition.

## Introduction: Exciting Embodiment

In the last two decades, cognitive science has embraced the thesis of “embodiment.” Embodied cognition stresses the intertwined nature of thinking and acting, and as such is an antidote to the traditional divide between cognition on the one hand and perception and action on the other. The excitement about embodiment within cognitive science lies mainly in its promise to destroy the traditional “sandwich” (or “hamburger”) model of cognitive processing, with its strict perception-cognition-action scheme (e.g., Hurley, 2001). The sandwich model regards “thinking” as the real stuff (the beef so to say), and takes perception and action as separated slave systems, providing input to cognitive processors (perception) and executing its commands (action).

Instead, embodied cognition stresses that perception and action are directly relevant for our thinking, and that it is a mistake to regard them as separate. The thesis comes in various formats, and a more in depth coverage is beyond the scope of this article (e.g., O’Regan, 1992; Van Gelder, 1995; Clark, 1997; Barsalou, 1999; Wilson, 2002; Noe, 2004; Gallagher, 2005; Wheeler, 2005).

In this paper we want to take stock and see what embodiment has done for a particular research domain in cognitive science, namely the study of semantic representations. With respect to semantic representations, embodied cognition is related to the claim of modality-specific versus abstract representations, in which modality-specific views predict sensori-motor cortex to be constitutive of conceptual representations (see Kiefer and Pulvermüller, 2012 for an excellent recent overview).

This being an opinion paper, it is by no means our intention to give an overview of the field. Instead we highlight certain studies, where we could have chosen others. Of particular importance is that we have chosen to ignore the neuropsychological literature regarding semantic representations (see e.g., Gainotti, 2000; Caramazza and Mahon, 2003; Kiefer and Pulvermüller, 2012).

## The Erosion of a Concept: The Case of Embodied Semantics Representations

Often embodied cognition is defined very broadly. When we for example look at experiments investigating “embodied semantics,” an important prediction is that understanding sensori-motor concepts leads to activation of sensori-motor cortices. So when people read about hand and foot actions, parts of the motor cortex involved in moving the hands and the feet are activated (e.g., Hauk et al., 2004; Tettamanti et al., 2005). Although interesting from the sandwich model perspective, it is unfortunate that the main hypothesis often does not go beyond predicting “involvement” of sensori-motor cortices (see also Binder and Desai, 2011; see also Chatterjee, 2010).

An illustration of this lack of specificity is how easily embodied cognition can capture strikingly different findings. For instance, Buccino et al. (2005) used single-pulse TMS to stimulate the hand or foot/leg motor area while participants were listening to sentences expressing foot and hand actions. Reaction times (RTs) and motor evoked potentials (MEPs) were specifically modulated for the effector involved in the described action: a hand-action-related sentence produced decreased MEPs in the hand area and slower RTs when subjects responded with their hand. The authors conclude that the processing of language modulates the activity of the motor system in an effector specific way. However, in another TMS study with a similar design Pulvermuller et al. (2005a) report that *faster* RTs are observed to hand/arm words after stimulation of the hand area.

It is striking that although the results are opposite (slower versus faster RTs), both are taken as confirmation of the embodied semantics theory. Instead, the researchers could have elaborated more about the reason of their divergent findings. For instance, maybe the differences arise because the interference occurs at a decision making level *after* semantic analysis (Mahon and Caramazza, 2008; Chatterjee, 2010). By formulating more specific hypotheses, e.g., here on the direction of the effect and the underlying mechanism, these findings could have been more informative. It strikes us as disappointing to not go beyond the conclusion of *involvement* of cortical motor areas; the pattern of results suggests that something more interesting is going on than motor cortex activation in response to action words. One is left with the question what result would be taken as evidence against embodied cognition?

Another sign of an underspecified theory is that similar findings can be interpreted as evidence in favor as well as against embodiment. Take the studies of Saygin et al. (2010) and Bedny et al. (2008).

First, Saygin et al. showed activation of perceptual (visual) areas when subjects were reading sentences describing motion. More specifically, they found increased BOLD levels in motion sensitive area MT+ when participants read sentences like “The wild horse crossed the barren field” versus “The black horse stood in the barren field” (Saygin et al., 2010). Second, in the study of Bedny et al. participants judged pairs of words that implied motion (animals, e.g., “the horse,” “the dog”), had intermediate implied motion (tools, e.g., “the sword,” “the axe”), or had little implied motion (natural kinds, e.g., “the bush,” “the pebble”). These authors did not find modulation of MT+ activity for words with different motion ratings. Regions within posterior lateral temporal cortex were more active when comparing verbs and nouns, independent of the amount of motion associations of the words.

A general theory of embodiment would have predicted both studies to find modulation in area MT+ related to amount of motion expressed in the materials. The fact that the one study does observe such modulation, and the other does not is an interesting clue to the context-dependence of sensory cortex activations during language comprehension or as Saygin et al. (2010) p. 2486) put it: “the choice of task and stimuli can influence the power to detect modulations of MT+ by linguistic events.” Instead, what happens is that one set of authors interpret their findings as in line with embodied cognition, and the other set of authors interprets their findings as evidence against embodiment, since they show that retrieval of sensory motor features is not obligatory during word comprehension (Bedny et al., 2008). The differences in their findings can probably be attributed to the differences in design. However, both studies generalize their results to the question of whether it supports an embodied or disembodied account, and it is in this interpretation stage that opposite conclusions are drawn.

Many experiments are driven by the “embodied versus disembodied” distinction. This is not a fruitful approach, and in the next section we will show that such a broad distinction does not do justice to the experimental findings that are available. To foreshadow our conclusion: Instead of quarreling about embodied versus disembodied, the field should take the next step and ask the question when and how sensori-motor cortices play a role in understanding.

## Taking Stock: Embodied Semantics

When we take a bird’s eye perspective toward experiments studying sensori-motor cortex involvement when participants read or listen to language describing sensori-motor events (action and visual language), a few things stand out:

– Sensori-motor cortices can be activated during language comprehension. For instance, cortical motor hand areas can be activated when participants read verbs related to hand actions (e.g., Hauk et al., 2004; Tettamanti et al., 2005).
– These sensori-motor activations can be fast (e.g., Pulvermuller et al., 2005b).
– Changing the activation level (via training or with TMS) of the motor system can influence processing of action-related language, suggesting a functional role (e.g., Glenberg et al., 2008; Willems et al., 2011).
– Some studies do not replicate sensori-motor activations when participants listen to action-language (e.g., Postle et al., 2008).
– Sensori-motor involvement is dependent on task and linguistic context (e.g., Sato et al., 2008; Papeo et al., 2009).

Of these findings, the latter one deserves more attention than it has gotten so far: Sensori-motor cortex involvement during understanding of action and perceptual language is task- and context-dependent.

For instance, it has been shown that the motor system is differently modulated depending on the experimental task. In a study by Sato et al. (2008) hand-action verbs interfered with button presses when participants performed a semantic task, but this was not the case when they performed a lexical decision task.

Similarly, in an elegant study Papeo et al. (2009) reported modulation of hand MEPs during reading of hand-action verbs when single-pulse TMS was applied, but again only during an explicit semantic categorization task (on action-relatedness) but not during a syllable detection task.

Another example of context-dependence is provided by Raposo et al. (2009) who showed that activation in motor cortex varied depending on the way verbs were presented: when verbs were viewed in isolation (“kick”) or in literal sentences (“kick the ball”) motor cortex was activated, but when the verbs were presented in idiomatic contexts (“kick the bucket”), no motor or premotor activation was present (see also Aziz-Zadeh et al., 2006; but see Boulenger et al., 2009).

Van Dam et al. (2012) varied the linguistic context in a different way: they instructed participants to focus either on the action or on the color aspect of a word’s referent. Activation in action- and motion-related areas was higher in the former than in the latter condition. The authors suggest that the “action” context emphasized action properties of the object and that therefore the corresponding action features were relevant in constituting the concept.

## Conclusion

So on the one hand, the state of affairs is favorable to embodied semantics: there can be involvement of sensori-motor cortices in understanding action and perceptual language. This is an important insight and definitely constitutes a way forward in our thinking about the neural basis of conceptual knowledge (see Kiefer and Pulvermüller, 2012 for overview). But the involvement of sensori-motor cortex in conceptual representations is of a more complex nature than a simple binary “yes” or “no.” Investigating “an involvement” of sensori-motor cortices in conceptual knowledge was perhaps a good first step, but needs to be followed up by more specific hypotheses. Future research needs to be more specific on when and how sensori-motor cortices are involved in language understanding. One reason for this is that current findings are too easily interpreted as confirming embodied accounts (see also Chatterjee, 2010). A second motivation is the fact that several studies show the context-dependence of sensori-motor involvement in language understanding. Computational models can be important in making the operations that take place in sensori-motor cortices more explicit, and the field should take more advantage of those (e.g., Chersi et al., 2010). Only with such specificity can embodied cognition make progress and will the concept retain its value.

## Conflict of Interest Statement

The authors declare that the research was conducted in the absence of any commercial or financial relationships that could be construed as a potential conflict of interest.
